# Prescription Intervals of Medications for Chronic Use: A Cohort Study

**DOI:** 10.1002/pds.70505

**Published:** 2026-10-16

**Authors:** Ryan Muddiman, Padraic Donoghue, Jessica Gómez Lemus, Ann Sinéad Doherty, Fiona Boland, Caroline McCarthy, Frank Moriarty

**Affiliations:** ^1^ School of Pharmacy and Biomolecular Sciences RCSI University of Medicine and Health Sciences Dublin Ireland; ^2^ Data Science Centre, School of Population Health RCSI University of Medicine and Health Sciences Dublin Ireland; ^3^ Department of General Practice RCSI University of Medicine and Health Sciences Dublin Ireland

**Keywords:** length bias, prescription interval, waiting time

## Abstract

**Purpose:**

In deprescribing studies, a prescription‐free gap is typically used to determine if patients discontinued their treatment. An appropriate gap depends on the typical time between prescriptions during continued use. This work aims to characterise the interval between prescriptions of chronic drugs (long‐term use) using different methods for a cohort of older people in primary care in Ireland.

**Methods:**

The prescription interval was analysed for 38 154 patients over 2011–2020 for the 20 most common drug classes in the dataset and the association between covariates and the interval was analysed using a multi‐level model. Estimates were also compared with those obtained from the parametric waiting time distribution (pWTD) approach.

**Results:**

Prescription and demographic factors had consistent relationships with prescription intervals across drug classes. Each additional prescription issue was associated with increases in the interval by 5.0 (NSAIDs) to 19.7 days (‘other antidepressants’). Full public health cover was associated with a −29.0 day (inhaled adrenergics) to −11.0 day (opioids) change relative to partial cover, whilst other/private health cover had a −17.9 day (benzodiazepines and associated drugs) to −7.1 day (SSRI and SNRIs) change relative to partial cover. The pWTD produced consistent estimates of the population interval for most drugs.

**Conclusions:**

The prescription interval varied substantially within drug classes, due to patient, practice and unmodelled factors. Variation between practices was effectively explained, with residual variation between patients and within patients. The pWTD approach is useful for describing complex distributions of intervals, and may be more appropriate for inferring a gap than summarising truncated data.

## Key Points

- Prescription intervals are highly focussed around intervals of typical prescribed quantities, as expected, with modes of approximately 30, 60 and 90 days for all drug classes.
- Heterogeneity in the prescription interval for specific drug classes was found to be mainly at the within‐patient level. This may be due to prescription renewal behaviour changing over time for individuals.
- Patient‐specific covariates, such as age, sex and healthcare scheme cover, had a similar effect on the prescription interval across the most frequent drug classes prescribed.
- The parametric waiting time distribution, using a finite mixture model to account for unmodelled variation, was able to robustly explain the population average distribution of empirical intervals using a penalised likelihood procedure.

## Plain Language Summary

The time between one drug prescription and the next prescription issued in general practice was calculated for the 20 most commonly prescribed drug classes in a database of older people attending primary care in Ireland. The timing varied drastically among drugs, due to differences in patients and practices. We analysed these variables using two approaches. The first looked at how factors such as age, sex and healthcare cover relate to the time between prescriptions. The second described the overall pattern of prescription intervals as a combination of simpler underlying patterns.

## 1 Introduction

There is a growing volume of research evaluating the occurrence and effects of deprescribing, defined as a clinician‐led intervention involving medication change with the goal of managing polypharmacy and improving outcomes. Routinely collected data, for example, prescribing data from an electronic health record (EHR) is a useful data source for this research [1]. The robustness of such research is contingent on accurately determining deprescribing exposure [2], and this is often done based on a static ‘gap’ following the last prescription [3]. However, the reality of healthcare delivery and patient behaviour (i.e., irregular healthcare visits and delays in requesting repeat prescriptions) can lead to variation in the timing of prescriptions which may impact on identifying cases of deprescribing using prescribing data. This variation, if not accounted for, may lead to exposure misclassification bias [4, 5]. Another major factor that can contribute to exposure misclassification is non‐adherence of prescribed or dispensed medication, however, adherence is unobservable in EHRs. In studies examining the effect of non‐randomised treatment strategies, the effect of this bias may be quantified using probabilistic bias analysis [6] however, such analyses require an estimate or distribution of the magnitude of misclassification, which is not available using standard gap‐based definitions of exposure. Furthermore, in a recent review of methodology within deprescribing studies, 27% of studies did not clearly report how they defined the date of last medication exposure [7]. Where a fixed gap was used, it also varied among studies even within drug classes [7]. Thus, analysing prescription intervals (the time between prescriptions) may reduce reliance on a generalisable ‘gap’ and could inform more accurate definitions of deprescribing [8].

Estimating prescription intervals using a representative population is one approach to defining periods of continued treatment and discontinuation [9]. The prescription interval can be obtained by measuring the time from one prescription issue until the next renewal. In the present study, this is named the empirical interval method. This method is restricted to those who renew at least once. A non‐parametric estimate provides a flexible summary of the distribution. When covariates are available, a more explanatory analysis can be performed by specifying a model for the intervals.

An alternative approach is to estimate the waiting time distribution (WTD) [9, 10]. The waiting time is the time from a calendar‐based index date (identical across the population) until the first observed prescription issue (see Figure 1). An estimate of the inter‐arrival density (IAD) (the probability density of renewal time for prevalent users) can be obtained from a parametric estimate of the WTD (pWTD), assuming the renewal process is stationary (i.e., the IAD is constant). The benefits over the empirical interval approach are the obviation of multiple renewals per patient and the potential to estimate the incidence of prescribing [11]. Using multiple random index dates has been shown to increase precision [12], and may average over nonstationary effects.

**FIGURE 1:**
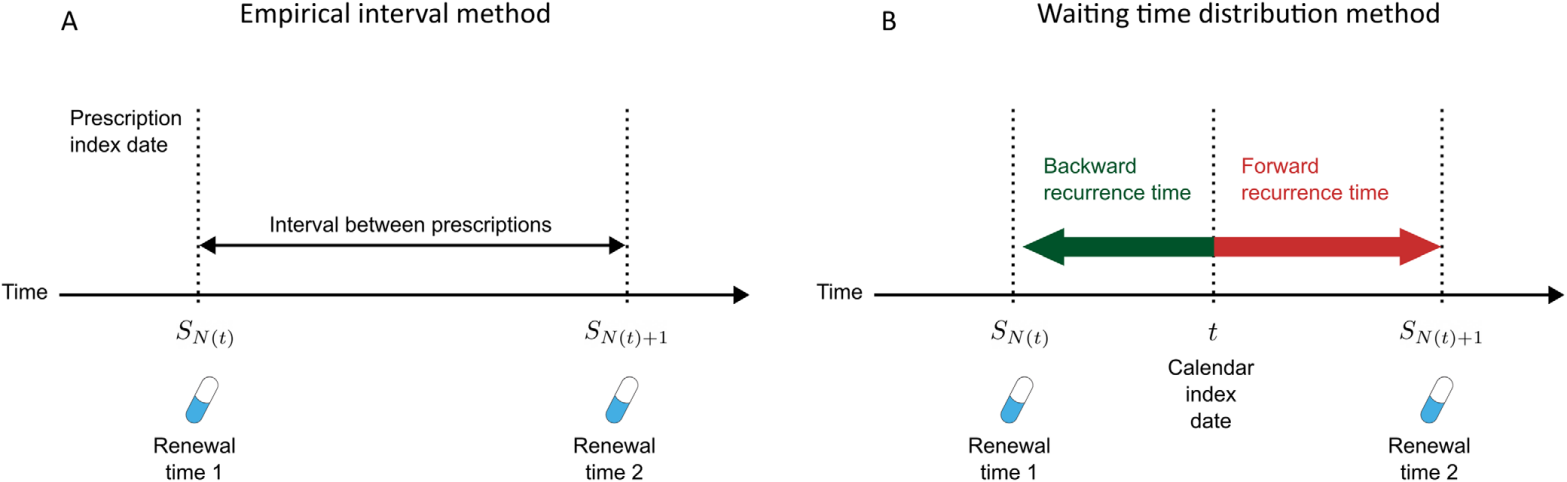
Schematic of the empirical interval method (A) and the waiting time distribution method (B) for prescription interval estimation.

This study aims to: (1) evaluate how prescription intervals (the time between successive prescriptions for a patient) in primary care vary across drug classes, patients and GP practices; and (2) compare estimates of the IAD of renewals using the pWTD approach and empirical interval approach.

## 2 Methods

A protocol was preregistered on the Open Science Framework [13] R version 4.5.0 was used for analysis [14].

### 2.1 Data Source

This study incorporates a secondary analysis of prescription data from a cohort of Irish patients from 44 GP practices [15]. Data was extracted for patients aged 65 years and older for the period 2011–2020, containing details of WHO Anatomical Therapeutic Chemical (ATC) codes and dates of prescribing, as well as information regarding patients' age, sex, number of prescription repeats and healthcare cover. Ireland has a mixed public‐private system of health cover, with approximately one‐third qualifying for full public health cover based on age and income [16].

### 2.2 Data Pre‐Processing

The 20 most frequently prescribed drug classes (excluding acute prescription drugs e.g., antibiotics) within the database were analysed. Prescriptions were classified into groups based on the fourth‐level ATC code (i.e., chemical subgroup), with some classes combined if they spanned more than one fourth‐level code for example, benzodiazepines (see S 1 of Supporting Information).

Covariates included age (continuous), sex (binary), health cover scheme (categorical: full public cover (General Medical Services scheme, providing medical services, prescription medicines and hospital care for free), partial public cover (Doctor Visit Card, providing free GP visits) and other/private cover: private cover and one other type), number of prescription repeats (integer) and anonymised GP practice and patient identifiers. The outcome was the interval between consecutive prescriptions. Intervals > 540 days (~18 months) were excluded as implausible within a single treatment course, with sensitivity analysis performed. This is effectively truncation and is a drawback of this method, which manifests due to conditioning on the presence of intervals, whereas the pWTD method does not condition on the presence of intervals. Prescriptions for the same drug on the same date had the number of issues set to the maximum value. Standardised scores were calculated for all intervals by subtracting the mean and dividing by the standard deviation, per drug class. Intervals with a magnitude of the score > 3 for continuous/ordinal variables were excluded via listwise deletion. Outlier removal was deemed necessary because there were highly implausible values of age, and sensitivity analyses were conducted. Continuous covariates were grand‐mean centred.

### 2.3 Analysis—Empirical Intervals

Prescription intervals were calculated and the marginal density estimated. Covariate associations were assessed using a multilevel linear model (MLM) with restricted maximum likelihood (REML) in *lme4* [17]. Two models were fitted, one including random intercepts only (unconditional mean model) as recommended, [18] and one including random intercepts and fixed covariate effects (full model). The full model was specified as

$$\begin{array}{c} {\text{Interval}}_{\mathrm{ij}}=\gamma_{0}+\beta_{1}\;{\text{repeats}}_{\mathrm{ij}}+\beta_{2}\;{\mathrm{age}}_{\mathrm{ij}}\\ \;+\beta_{3}\;{\mathrm{sex}}_{\mathrm{ij}}+\beta_{4}\;{\text{cover}}_{\mathrm{ij}}+u_{\text{Practice}\left(i\right)}+u_{\text{Patient}\left(j\right)}+\varepsilon_{\mathrm{ij}} \end{array}$$

and the mean model as follows:

$${\text{Interval}}_{\mathrm{ij}}=\gamma_{0}+u_{\text{Practice}\left(i\right)}+u_{\text{Patient}\left(j\right)}+\varepsilon_{\mathrm{ij}}$$

where γ_0_ is the grand mean intercept, *β*_*i*_ is the coefficients, ${\text{repeats}}_{\mathrm{ij}}$ is the number of prescription repeats, ${\text{cover}}_{\mathrm{ij}}$ is the healthcare cover type, $u_{\text{Practice}\left(i\right)}$ is the GP practice random effect, $u_{\text{Patient}\left(j\right)}$ the patient random effect and $\varepsilon_{\mathrm{ij}}$ is the error term.

### 2.4 Analysis—Waiting Time

The WTD in this context is the distribution of time from an index date D to the next prescription renewal $R_{i}$ for all patients [19]. The WTD density *h(R*_*i*_ *‐D)* can be modelled as

$$h\left(R_{i}-D\right)={\mathrm{wf}}_{\mathrm{rec}}+\left(1-w\right)f_{\mathrm{inc}}$$

where $f_{\mathrm{rec}}$ is a forward recurrence density (FRD), $f_{\mathrm{inc}}$ is an incidence density and w is the proportion of prevalent patients (see Supporting Information for details). When the renewal process is in equilibrium (the time origin of the process is in the distant past), $f_{\mathrm{rec}}$ is a function of $f_{\mathrm{IAD}}$ only [20].

$$f_{\mathrm{rec}}=\frac{1-\int_{-\infty }^{x}f_{\mathrm{IAD}}}{\mu }$$

where *μ* is the mean of $f_{\mathrm{IAD}}$. Thus, a parametric form can be assumed for $f_{\mathrm{IAD}}$ and $f_{\mathrm{inc}}$, and $h\left(R_{i}-D\right)$ can be fit to the data. The IAD can then be obtained by substitution of point estimates of the parameters of the distribution into the analytic equation. This method has previously been implemented using the maximum likelihood estimation (MLE) procedure assuming a two‐component mixture of recurrence densities and a single uniform incidence density [11]. In this work, the model was extended to an arbitrary but finite number (*g*) of recurrence densities. This was due to the added flexibility of modelling a larger number of clusters, due to package sizes or typical prescription durations [11]. Ten random index dates were used and *g* was varied from 1 to 5. All $f_{\mathrm{IAD},i}$ were chosen to be from the lognormal family due to its non‐negative support. The Bayesian Information Criterion (BIC) was used to find the optimal *g* value [21] and the Akaike Information Criterion (AIC) was also reported (see Supporting Information for details). After obtaining the optimal *g* (best of 5 runs), a separate model was fit again to obtain the pWTD parameters. The code is on Zenodo [22].

## 3 Results

Overall, 38 154 patients who were prescribed one of the included long‐term drug classes were examined, with a mean age of 78 years and 43% were male (see Table 1). Across the included drug classes, 2.65 million renewals occurred, with a median of 3 prescription repeats (IQR 1–3). The proportion of intervals exceeding 18 months was 1.7% of all intervals. The number of issues stratified by drug class is shown in the S 2 of Supporting Information. The level of missingness for age was 0.45% and 0.43% for sex. The number of issues and healthcare scheme had no missing values. Due to this, imputation was not performed. Statins, proton pump inhibitors (PPIs) and paracetamol were the three most common drug classes prescribed.

**TABLE 1:** Data characteristics after pre‐processing.

Characteristic	*N* (%)^a^
Patient characteristics (*n* = 38 154)	
Age (years), mean (SD)	78 (9)
Sex	
Female	21 628 (57%)
Male	16 526 (43%)
Health cover type	
Partial public cover (Doctor Visit Card)	4111 (11%)
Full public cover (General Medical Services scheme)	26 574 (70%)
Other/private	7469 (20%)
Prescription characteristics (*n* = 2 652 118)	
Prescription repeats, median (IQR)	3.00 (1.00, 3.00)
Drug	
Statins	365 633 (14%)
Proton pump inhibitors	287 605 (11%)
Paracetamol	219 542 (8.3%)
Angiotensin receptor blockers ± diuretics	207 211 (7.8%)
Beta blockers	199 478 (7.5%)
Calcium ± vitamin D	171 724 (6.5%)
Antiplatelets (incl. low dose aspirin)	162 441 (6.1%)
Benzodiazepines and associated drugs	151 175 (5.7%)
ACE inhibitors ± diuretics	122 666 (4.6%)
DHP calcium channel blockers ± diuretics	113 516 (4.3%)
Laxatives	103 304 (3.9%)
SSRIs and SNRIs	103 020 (3.9%)
Inhaled adrenergics	80 245 (3.0%)
Inhaled adrenergics with corticosteroids and/or anticholinergics	75 062 (2.8%)
Thyroid hormones and derivatives	66 004 (2.5%)
NSAIDs	54 236 (2.0%)
Opioids	50 436 (1.9%)
Oral anticoagulants	46 187 (1.7%)
Gabapentinoids	38 886 (1.5%)
Other antidepressants	33 747 (1.3%)

Abbreviations: ACE: angiotensin‐converting enzyme; DHP: dihydropyridine; NSAID: non‐steroidal anti‐inflammatory drug; SNRI: serotonin and norepinephrine reuptake inhibitor; SSRI: selective serotonin reuptake inhibitor.

^a^ Unless otherwise stated.

### 3.1 Empirical Distribution and Multi‐Level Modelling

The number of prescription interval pairs per drug class varied from 365 633 (statins) to 33 747 (‘other antidepressants’). The marginal IAD is shown in Figure 2A. The graph shows a multi‐modal structure with modes spaced by approximately 1 month (as expected due to the typical intended prescription durations). Beyond 120 days, there were low numbers of renewals across all drug classes, potentially due to different treatment episodes. The intervals were stratified by the number of prescription repeats, and multi‐modality persisted (S 3). The marginal distribution is shown in S 4 of Supporting Information.

**FIGURE 2:**
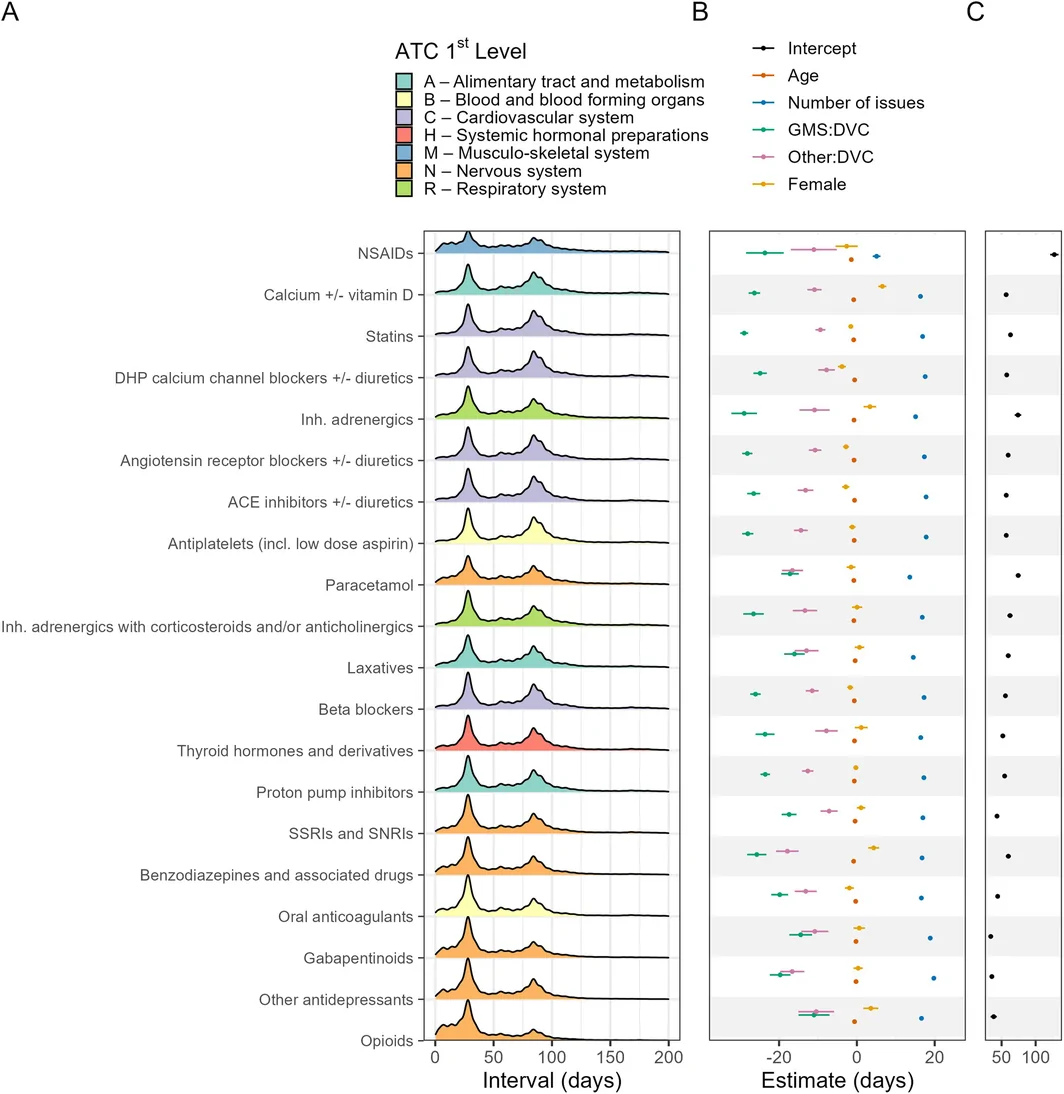
(A) Kernel density estimate of the inter‐arrival distribution for all drug classes. The drugs are ordered by mean interval descending. (B) Coefficients and (C) population‐level intercept for the multi‐level model. ACE: angiotensin‐converting enzyme; DHP: dihydropyridine; DVC: partial public cover; GMS: full public cover; Inh.: inhaled; NSAIDs: non‐steroidal anti‐inflammatory drugs.

Figure 2B shows the fixed effects coefficients and grand mean intercept (across‐level mean interval when covariates are equal to the reference value) with their 95% confidence intervals obtained from the full model. The grand mean intercept varied substantially, from 34.0 days (gabapentinoids) to 127.4 days (NSAIDs), as seen in Figure 2B. For 10 drug classes, the intercept was in the range of 50–60 days. The number of prescription repeats was associated with an increase in the prescription interval by 5.0 days (NSAIDs) to 19.7 days (‘other antidepressants’), per additional prescription issue. A patient being female was associated with a −3.9 day (dihydropyridine calcium channel blockers ± diuretics) to 6.5 day (calcium ± vitamin D) change relative to being male. A 5‐year difference in age was associated with a −7 day (NSAIDs) to −1.0 day (‘other antidepressants’) change in the interval. Finally, full public cover was associated with a −29.0 day (inhaled adrenergics) to −11.0 day (opioids) change relative to partial public cover patients, whilst those with other/private cover had an associated −17.9 day (benzodiazepines and associated drugs) to −7.1 day (SSRI and SNRIs) change relative to partial public cover scheme patients.

The variance at each level for the null and full models is shown in Figure 3. The distribution of the random intercepts is shown in S 5. The MLM results without outlier removal and after truncating at a shorter gap of 270 days are also shown in S 6–S 8.

**FIGURE 3:**
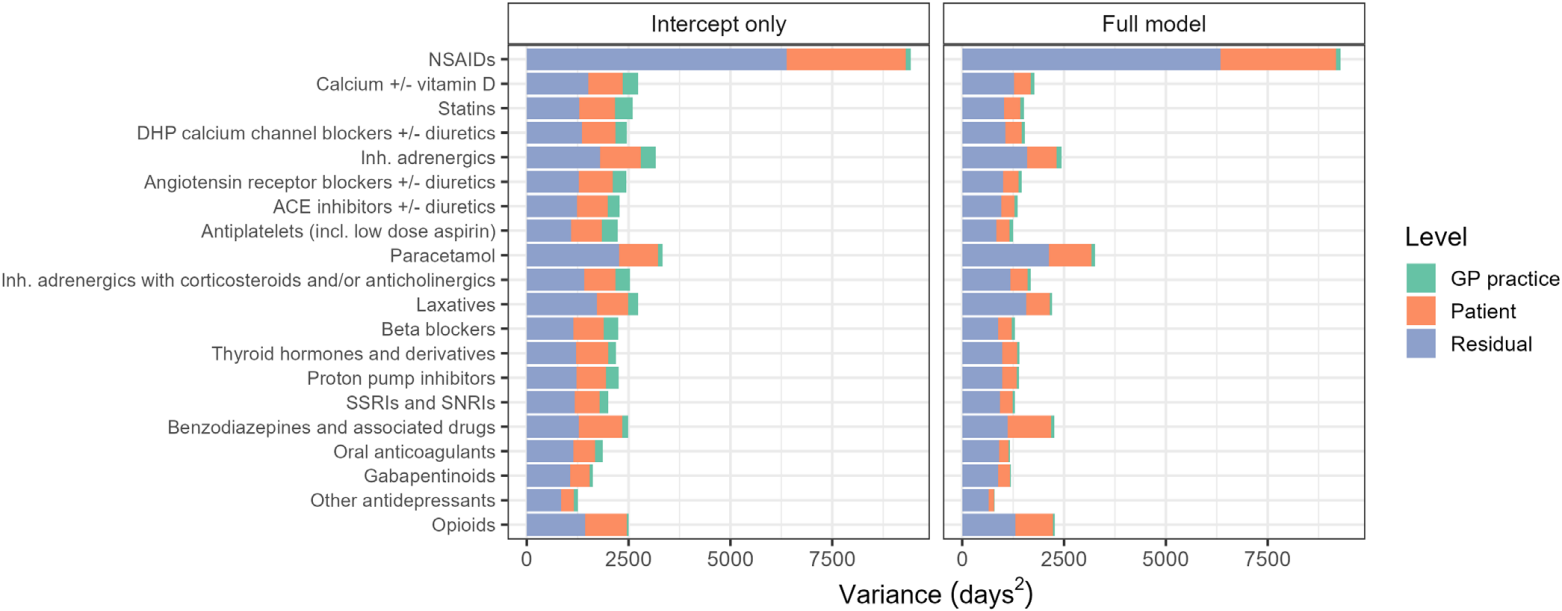
Variance of each model by group level.

### 3.2 Parametric Waiting Time Distribution

The model selection results are shown in Figure 4A, plotting the BIC and AIC versus the number of FRD components. This showed that 3 was the optimal value of *g* for 10 of the classes. The results of the fitting procedure are shown in Figure 4B using QQ plots. The median and 80th percentile for the empirical and modelled IAD is shown in Table 2, which generally showed good agreement between empirical and fitted densities over the chosen quantiles, with NSAIDs, angiotensin receptor blockers ± diuretics, antiplatelets (incl. low dose aspirin), paracetamol, beta blockers, SSRIs and SNRIs and oral anticoagulants classes being an exception.

**FIGURE 4:**
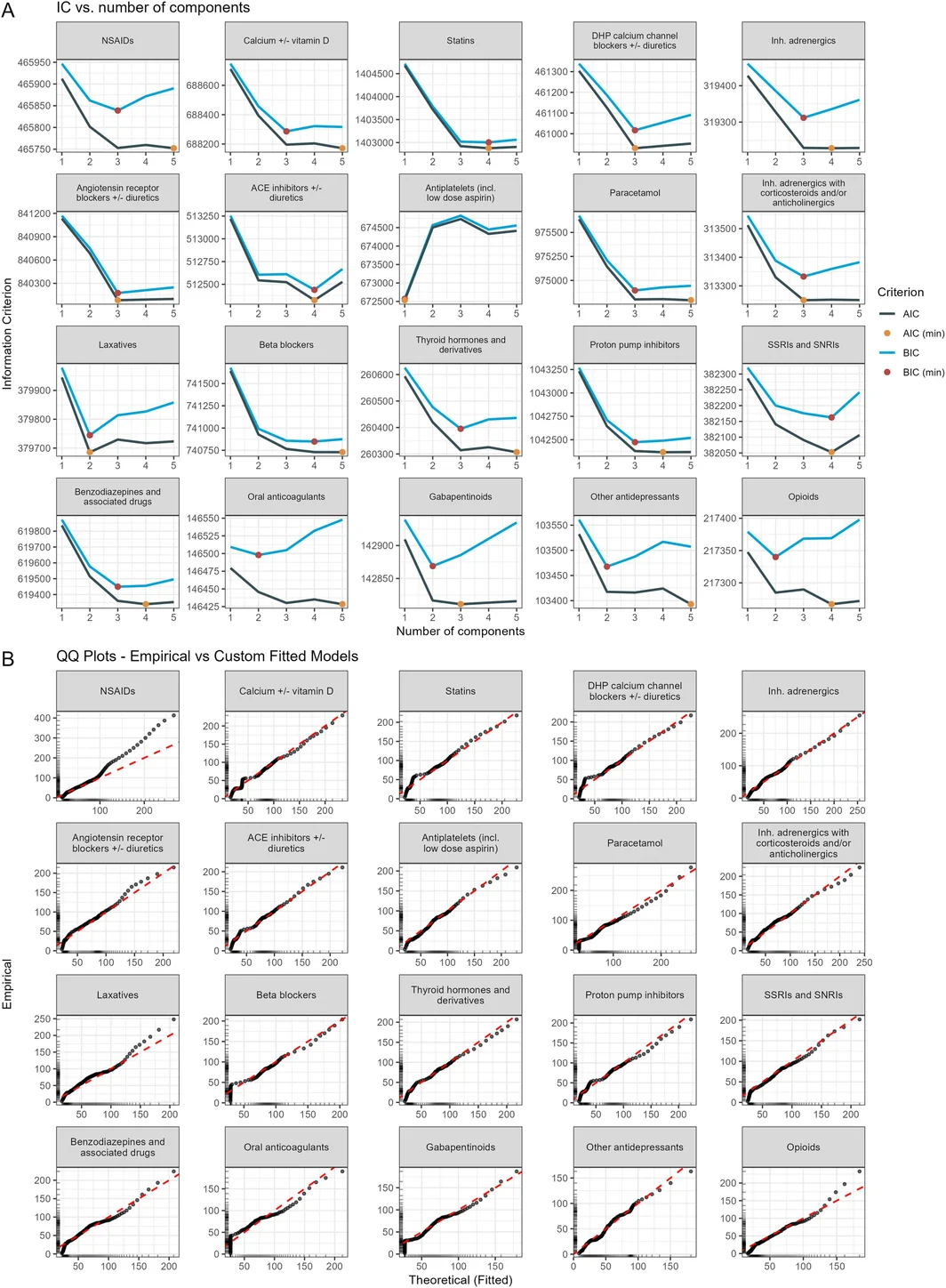
(A) AIC and BIC with their minima labelled versus the number of mixture components stratified by drug class, (B) QQ plots of the empirical versus model‐based distribution stratified by drug class.

**TABLE 2:** Empirical and fitted inter‐arrival density values at the 0.5 and 0.8‐quantile.

Drug	Quantile			
	0.5		0.8	
	Empirical	Fitted (95% CI)	Empirical	Fitted (95% CI)
NSAIDs	77	71.3 (67.4–75)	149	112.1 (107.2–120.8)
Calcium ± vitamin D	76	78.1 (76.2–79.4)	103	102.1 (98.9–105)
Statins	77	74.6 (73.2–75.6)	100	99.7 (98.7–100.4)
DHP calcium channel blockers ± diuretics	77	74 (71.6–76.5)	100	100.1 (98.1–102.1)
Inh. adrenergics	69	64.2 (58.1–72.1)	100	98.7 (96.2–101.8)
Angiotensin receptor blockers ± diuretics	76	71.6 (70–74.1)	98	95 (93.8–95.9)
ACE inhibitors ± diuretics	75	72.7 (71.4–73.8)	97	96.3 (95.1–97.7)
Antiplatelets (incl. low dose aspirin)	76	73.5 (71.6–75.1)	97	99.9 (98.5–101.2)
Paracetamol	63	68.4 (65.4–70.5)	98	106.2 (103.4–110.7)
Inhaled adrenergics with corticosteroids and/or anticholinergics	67	60.7 (55.8–66.2)	98	99.9 (97.6–102.2)
Laxatives	66	56.4 (53.9–59.5)	96	98.2 (95.8–101.4)
Beta blockers	71	75.4 (73.5–77.5)	95	98.6 (97.2–99.7)
Thyroid hormones and derivatives	67	68.9 (65.2–72.2)	95	97.5 (95.2–99.6)
Proton pump inhibitors	65	67.9 (66.7–69.2)	94	96.6 (94.7–99)
SSRIs and SNRIs	56	62.1 (57.9–66)	91	96.4 (94–98.1)
Benzodiazepines and associated drugs	50	49.4 (46.8–52.1)	90	94.8 (93.2–96.4)
Oral anticoagulants	46	35.4 (35.2–36.8)	88	93.4 (93.3–94.4)
Gabapentinoids	39	47.7 (44.3–49.3)	86	86.6 (84.2–88.8)
Other antidepressants	42	46.8 (43.9–50.5)	85	87.8 (86.2–90.5)
Opioids	31	35.4 (34–37.5)	83	82.6 (78.8–86.6)

*Note:* Confidence intervals are pointwise, based on 20 bootstrap samples.

Abbreviations: ACE: angiotensin‐converting enzyme; DHP: dihydropyridine; Inh.: inhaled; SNRI: serotonin and norepinephrine reuptake inhibitor; SSRI: selective serotonin reuptake inhibitor.

## 4 Discussion

The most frequent intervals for all drug classes were centred around 30 and 90 days, consistent with a similar study in England [23]. There was also markedly similar shapes in the marginal distributions between drug classes. The extended intervals in the right tail of the interval distribution may be due to an increased quantity of medication being prescribed at the previous visit, for example when anticipating travel, different treatment courses for some drugs, or prescriptions given during hospitalisation. There was a distinct probability mass around < 25 days for NSAIDs, other antidepressants, benzodiazepines, gabapentinoids and opioids. These may be due to extending short courses of treatment, or smaller quantities being prescribed due to risk of misuse. The potential impact is bias in the descriptive estimates of the interval. For example, short‐term prescribing may have a systematically higher variance in the interval compared with chronic prescribing due to intermittent use. Therefore, the inter‐arrival distribution and other derived statistics may be different compared with exclusively analysing intervals from intended long‐term use. A shorter prescription duration (e.g., 1 month) may be typical for initial prescriptions of a medicine. However, with ongoing use, these intervals would contribute much less compared with those intervals corresponding to chronic prescribing after tolerance has been confirmed. The fixed effect coefficients were largely unchanged for the smaller choice of interval truncation (see S 7 and S 8), but the intercepts and variance were reduced. The residual variance of the NSAID class was also more comparable with the rest of the drug classes for this case, suggesting that intervals beyond 270 days significantly contributed to the original residual variance for this class (likely repeated acute use). The MLM exhibited heteroskedasticity and nonlinear residuals and the standard errors of the fixed effects were expected to be biased as a result of the misspecification due to omitted terms and/or parametric assumptions being violated. Therefore, no hypothesis tests were conducted.

We found that the variance was largest at the residual level (see Figure 3), that is, neither at the between‐practice or between‐patient level, given the predictors available. Due to the limited demographic information available, there was no conclusive evidence that patient factors may explain this variance, but we can conclude that this variation is not explained by factors correlated with GP practice or by the available demographics. We hypothesise that unmodelled factors such as socioeconomic status, health literacy and baseline health status, which were not captured in the data, may explain this variation. High within‐patient variation in the interval may result in misclassification of exposure and large numbers of identified intercurrent events such as restarting. The change in the total variance when patient covariates were included, as shown in Figure 3 and tabulated in S 9, shows that the covariates explain some of the variation, however the change in total variance was negligible for some drug classes such as paracetamol and NSAIDs. This could be due to latent unmeasured variables at patient or practice level, such as disease status. The GP practice‐level variance decreased for all drug classes compared with the null model. This was likely due to a different distribution of characteristics of patients between practices, which may be associated with the prescription interval. The multi‐modal nature of the marginal IAD was partly explained by the number of prescription repeats (i.e., individual repeats per prescription). The residual variance (shown in Figure 3) may be due to deviations in renewal behaviour, such as re‐issues before the end of supply.

The model selection results for the pWTD show that the optimal *g* (minimising BIC) was between 1 and 5, with 10 of the drug classes having an optimal of 3. The number of components may correspond with latent subpopulations that share a common characteristic, such as prescribed duration of the drug, or from systematic differences for example, default short‐term supplies for first‐time prescriptions versus longer supplies for chronic use. A model using log‐transformed intervals had practically no difference in the fixed effects coefficients (S 10). An example parametric fit for statins is shown in the S 11 of Supporting Information. For all but one class, the BIC curve was convex, highlighting that the range of *g* was appropriate. Since the likelihood surface may be non‐convex in a mixture model, the choice of starting values for the maximum likelihood optimisation may have resulted in a local minimum being found [24]. Therefore, increasing the number of replications for the optimisation may have resulted in a different value of *g* for that drug class.

The pWTD and empirical distribution had good agreement for the majority of drug classes as seen in Figure 4B. Table 2 also shows very close agreement between the empirical and fitted median IAD. There were two noticeable regions of lack‐of‐fit. Firstly, in the right tail there was systematic over‐estimation of the IAD for certain classes for example, antiplatelets (incl. low dose aspirin), paracetamol, beta blockers and oral anticoagulants. This was also apparent for the 80th percentile in Table 2. This may be due to the lack of data at the longer intervals. The pWTD has previously been validated for warfarin [25] We considered a broader range of oral anticoagulants, combining warfarin, and so forth with others which may be the cause of the lack of fit. When determining discontinuation times for drugs with chronic and non‐chronic use (e.g., NSAIDs) within a cohort study, this problem may resolve because inclusion criteria typically exclude those with short medicine usage. The other feature in the QQ plots was a non‐smooth trend at approximately 30 days in the calcium ± vitamin D, statins, DHP calcium channel blockers ± diuretics, Paracetamol, beta blockers, PPIs, oral anticoagulants and gabapentinoids classes. This was likely due to distributional assumptions being violated. Figure 2A shows significant probability mass in the 0–30‐day range that would not be well described by a lognormal distribution. Other distributions such as a Weibull distribution could be explored, which may produce better fit in this region. When the MLM was fit without outlier removal, the precision of the fixed effects estimates decreased due to larger variance in the response (see S 6). The intercept also had substantial variation compared with the pre‐processed data. However, the fixed effects did not differ drastically from the pre‐processed results.

A limitation of this work is the restriction to Irish primary care data from older adults, which may not be generalisable to other countries or patient groups. Different countries may have varying clinical guidelines and prescribers may issue chronic prescriptions at different intervals. Therefore, replication of this work using prescription databases from other healthcare contexts would be beneficial. Conditioning on the presence of at least two prescriptions within a prespecified time window was done in order to limit the analysis to prevalent users. A limitation is that termination of the renewal process or administrative censoring was not accounted for. The process may terminate due to deprescribing, death, patient‐led decisions (non‐adherence or change of provider) or other factors. Estimating the time when termination occurs in an observational database is difficult, since no information is typically recorded when this occurs [26]. The IAD can be used to extrapolate a ‘stopping time’ estimate using a decision rule. When applied to chronic medications there is a risk of underestimating the true stopping time due to stockpiling and non‐adherence. Thus, a sensitivity analysis is recommended [27]. A decision rule based on the IAD for defining exposure is a crude method that should be used when no other patient‐ or prescriber‐related information is available.

## 5 Conclusions

This study analysed prescription intervals for the most frequently prescribed drugs in a GP database of older adults in Ireland. The interval was highly variable within drug classes and the variation was mostly at patient‐level and was not adequately explained by the measured covariates. This study should contribute to improved research practices in observational studies examining deprescribing or discontinuation. Such studies often rely on decision rules to determine exposure status from prescriptions, termed gap‐based methods. This work enables researchers to incorporate data‐informed estimates of intervals in their research rather than using arbitrary gaps, potentially enhancing the classification accuracy. Across all drug classes, the fixed effects of patient/prescription characteristics had consistent direction and magnitude of association with the renewal time, suggesting that the covariates exerted a similar influence on the interval regardless of the type of drug prescribed. Consequently, dynamic gaps are unlikely to provide additional benefit.

The parametric waiting time distribution is useful for characterising prescription intervals without conditioning on prevalent users. Incorporating mixtures of distributions allows modelling of the complex interval distribution. When implementing a population‐based decision rule to define medication discontinuation, such as one derived from the marginal pWTD, substantial prediction error may remain at the individual level. However, compared with simpler single‐distribution or conditional approaches, mixture models may provide more accurate estimation of the population renewal‐time distribution and therefore provide better‐calibrated static discontinuation measures, as they account for heterogeneity in renewal behaviour.

## Funding

This work was supported by Wellcome Trust (227348/Z/23/Z) and Irish Health Research Board (CSF‐2023‐012).

## Ethics Statement

This study was approved by the Irish College of General Practitioners Research Ethics Committee.

## Conflicts of Interest

The authors declare no conflicts of interest.

## Supporting information


**Data S1:** Supporting Information.
**S 1**. Anatomical Therapeutic Chemical (ATC) groupings.
**S 2**. Number of issues stratified by drug class.
**S 3**. Marginal empirical interval stratified by drug class and number of prescription repeats.
**S 4**. Marginal empirical distribution functions for each drug class.
**S 5**. (a) Boxplot of the distribution of practice‐level random intercepts (b) boxplot of the distribution of patient‐level random intercepts.
**S 6**. Fixed effects from the multi‐level model without covariate outlier removal.
**S 7**. Fixed effects from the multi‐level model with intervals truncated at 270 days.
**S 8**. Variance by level with intervals truncated at 270 days.
**S 9**. Model variance by drug class, model and level.
**S 10**. Fixed effects from the log‐transformed multi‐level model.
**S 11**. Waiting time distribution and parametric fit results for statins.

## Data Availability

Data sharing not applicable to this article as no datasets were generated or analysed during the current study.
